# Development of an IL-17A DNA Vaccine to Treat Systemic Lupus Erythematosus in Mice

**DOI:** 10.3390/vaccines8010083

**Published:** 2020-02-12

**Authors:** Hiroshi Koriyama, Yuka Ikeda, Hironori Nakagami, Munehisa Shimamura, Shota Yoshida, Hiromi Rakugi, Ryuichi Morishita

**Affiliations:** 1 Department of Health Development and Medicine, Osaka University Graduate School of Medicine, Suita, Osaka 565-0871, Japan; 2 Department of Clinical Gene Therapy, Osaka University Graduate School of Medicine, Suita, Osaka 565-0871, Japan; 3 Department of Geriatric and General Medicine, Osaka University Graduate School of Medicine, Suita, Osaka 565-0871, Japan

**Keywords:** IL-17A, vaccine, SLE

## Abstract

The interleukin-17 (IL-17) family, especially IL-17A, plays an important role in the pathogenesis of systemic lupus erythematosus (SLE). This study developed an IL-17A epitope vaccine to treat SLE in NZBWF1 and MRL/lpr mouse models. A plasmid vector encoding a hepatitis B core (HBc)-IL-17A epitope fusion protein was injected using electroporation into the skeletal muscle of NZBWF1(New Zealand Black mice x New Zealand White mice F1 hybrid strain) or MRL/lpr mice three times at 2-week intervals. As a result, anti-IL-17A antibodies were successfully produced in the HBc-IL-17A group. Accordingly, serum tumor necrosis factor alpha (TNF-α) concentrations were significantly reduced in the HBc-IL-17A group. According to pathological analysis, the IL-17A DNA vaccine significantly suppressed renal tissue damage and macrophage infiltration. Consequently, the survival rate was significantly improved in the HBc-IL-17A group. In addition, we evaluated the antigen reactivity of splenocytes from IL-17A-immunized mice using an enzyme-linked immune absorbent spot (ELISPot) assay for safety evaluation. Splenocytes from IL-17A-immunized mice were significantly stimulated by the HBc epitope peptide, but not by the IL-17A epitope or recombinant IL-17A. These results indicate that the IL-17A vaccine did not induce autoreactive T cells against endogenous IL-17A. This study demonstrates for the first time that an IL-17A DNA vaccine significantly reduced organ damage and extended survival time in lupus-prone mice.

## 1. Introduction

Systemic lupus erythematosus (SLE) is a prototypic systemic autoimmune disease characterized by abundant autoreactive CD4^+^ T cells, excessive autoantibody production, and immune complex deposition, with highly heterogeneous clinical manifestations [1,2]. Lupus nephritis is a severe inflammatory disease of the kidneys caused by systemic SLE. Although the detailed and specific pathogenesis is not completely understood, several recent reports have indicated that the cytokine interleukin-17A (IL-17A) and its related T helper cell subset, Th17, play critical roles in the pathophysiology of SLE [3,4]. From clinical studies, the serum IL-17A concentration in SLE patients has been reported to be significantly higher than that of controls [5,6,7]. Based on these findings, it is hypothesized that type I interferon and IL-17A would form a feedback loop and play an important role in the progression of SLE [4]. As anti-IL-17A antibody therapy, we developed a DNA vaccine in this study to induce an anti-IL-17A antibody to treat SLE, since our previous reports successfully documented the development of therapeutic B cell vaccines to neutralize autoantibodies against targets such as angiotensin II [8] and DPP (Dipeptidyl Peptidase)-IV [9].

Here, we successfully developed an IL-17A DNA vaccine to treat SLE in two SLE models, NZBWF1 (F1 hybrid strain between New Zealand Black and New Zealand White mice) and MRL/lpr mice.

## 2. Materials and Methods

### 2.1. Vaccine Design and Synthesis

Based on the three-dimensional structure of the IL-17A protein, we selected 2 epitopes as candidate vaccine targets: 17A1 (amino acid (aa) 65–72) and 17A2 (aa 110–116). The construction of a hepatitis B core (HBc)-fusion epitope DNA vaccine was described previously [8]. Briefly, we used plasmid pcDNA3.1 (pcDNA3.1/V5-His-TOPO, Invitrogen) containing the cytomegalovirus promoter. The HBc gene was obtained by PCR using pPLc3 (BCCM/LMBP, Belgium) as the template and was ligated into pcDNA3.1. The construct (pcDNA3.1-HBc) was used as a control HBc vaccine. To construct pcDNA3.1-HBc-IL-17A, PCR was performed 3 times. First, one fragment contained the sequence of the spacer Ile-Thr dipeptide and the N-terminal half of IL-17A epitope sequence was amplified by using a primer for the 5′ half of the HBc ORF (HBc-N). Second, another fragment contained the sequence of the C-terminal half of IL-17A and the tripeptide spacer Gly-Ala-Thr was amplified by using a primer for the 3′ half of the HBc ORF (HBc-C). Using these 2 amplified fragments as templates, a third PCR was performed using primers HBcF and HBcR [8]. The final amplified fragment contained the IL-17A epitope sequence within the immunodominant region (aa 80–81) of HBc.

### 2.2. Mice

To select an appropriate antigen for the IL-17A vaccine, 6-week-old female BALB/c mice were used in this study. To evaluate the efficacy of the IL-17A vaccine in the spontaneous SLE mouse model [10], 6–8-week-old female NZBWF1 and MRL/lpr mice were used in this study. The experimental protocols were approved by the Ethical Committee for Animal Experiments of the Osaka University Graduate School of Medicine (27-020-032) and performed in accordance with the National Institutes of Health (NIH) Guidelines for the Care and Use of Laboratory Animals.

The animals were kept in a room with controlled lighting (12 h light/dark cycle), pressure, humidity, and temperature (24 °C) with free access to food and water.

### 2.3. DNA Immunization

All mice were purchased from Oriental Yeast (Osaka, Japan). After anesthesia by isoflurane, mice were given a 60 μL vaccination intramuscularly containing 120 μg of plasmid DNA or saline using an electric pulse generator with a pair of stainless steel needles. The needles were 10 mm in length and 0.3 mm in diameter, with a 3 mm fixed distance between them (Nepa Gene Co., Ltd., Ichikawa, Japan). The voltage remained constant at 70 V during the pulses. Three pulses at the indicated voltage followed by 3 more pulses of the opposite polarity were administered to each injection site at a rate of 1 pulse/s, each pulse 50 ms in duration.

### 2.4. Anti-IL-17A Antibody Titer Assay

ELISA was performed as previously described. Briefly, bovine serum albumin (BSA) conjugated IL-17A1 (AF-588-AB1, lot no. 2839-AB1) and IL17A2 (AF-589-AB1, lot no. 2841-AB1) were synthesized at their N-terminus by suberic acid bis (Peptide Institute Inc., Osaka, Japan). The BSA-IL-17A epitope was coated onto ELISA plates (MaxiSorp Nunc, Thermo Fisher Scientific K.K. Tokyo, Japan) at 10 μg/mL in carbonate buffer overnight at 4 °C. After blocking with a 5% skim milk solution in phosphate-buffered saline (PBS), serial dilutions (1:10–1:31,250) of serum samples from the immunized mice were added to the wells and incubated at 4 °C overnight. After washing with PBS (Phosphate Buffered Saline)–0.05% Tween (PBS-T), the plate was incubated with horseradish peroxidase (HRP)-conjugated anti-mouse IgG (GE Healthcare, Tokyo, Japan) for 3 h at room temperature. After washing, the color was developed using 3, 3′, 5, 5′-tetramethylbenzidine (TMB) solution (Sigma-Aldrich, Tokyo, Japan), and the reaction was stopped using 0.5 N sulfuric acid. The absorbance was read using a microplate reader (Bio-Rad Inc., Hercules, CA). The endpoint titer was expressed as the serum dilution that exhibited half-maximal binding.

### 2.5. Immunoblotting

Recombinant mouse IL-17A (421-ML-0251CF, R&D Systems), BSA-IL-17A1 conjugate, and BSA-IL-17A2 conjugate were separated electrophoretically using SDS/PAGE and blotted onto poly(vinylidene difluoride) membranes (Millipore). The blots were incubated with sera from mice immunized with the IL-17A1 or IL-17A2 vaccine, an anti-BSA antibody, or a commercially available anti-IL-17A antibody (AF-421-NA, R&D Systems). After incubation with HRP-conjugated antibodies specific for mouse IgG (GE(general electrics) Healthcare, Hino, Japan), the chemiluminescence signal was detected using a Fujifilm LAS 1000 camera (Fujifilm, Tokyo, Japan) and analyzed using MultiGauge version 3.2 software (Fujifilm, Tokyo, Japan).

### 2.6. Measurement of Serum Cytokines

Serum IL-17A (cat. no. M1700), IL-1β (cat. no. MLB00C), and tumor necrosis factor alpha (TNF-α; cat. no. MTA00B) concentrations were measured using ELISA (Quantikine, R&D Systems, Minneapolis, MN, USA).

### 2.7. Histological Examination

For immunohistochemistry (IHC) analysis, isolated kidney was fixed in 4% paraformaldehyde for 24 h, embedded in paraffin, and sliced in 4 μm sections. The sections were incubated with a primary antibody (rat monoclonal anti-F4/80 antibody, 1:200 dilution; cat. no. ab6640, Abcam) and a secondary antibody (biotinylated anti-rat IgG, Vector Laboratories, Burlingame, CA). For F4/80 immunostaining, sections were counterstained with hematoxylin and mounted for microscopic observation (Olympus FSX100, Tokyo, Japan). For histological examination, mouse liver, kidney, spleen, and submandibular glands were dissected, fixed in 4% paraformaldehyde overnight at 4 °C, and then embedded in paraffin. Specimens were sliced into 4 μm thick sections and stained with hematoxylin and eosin (H&E) or periodic acid Schiff (PAS).

### 2.8. ELISpot Assay

These assays were performed as described previously [8]. Briefly, 96-well enzyme-linked immune absorbent spot (ELISpot) plates (Millipore, Tokyo, Japan) were coated with an anti-mouse interferon gamma (IFN-γ) or anti-mouse IL-4 capture antibody overnight at 4 °C. After incubation, the plates were washed with PBS containing 0.05% Tween 20 (PBS-T) solution and then blocked with 1% BSA and 5% sucrose in PBS. Splenocyte suspensions from immunized mice were added to the plates (10^6^ cells/well) and stimulated with 10 μg/mL IL-17A1 peptide, 17A2 peptide, recombinant mouse IL-17A, HBc peptide, or phytohemagglutinin (PHA) at 37 °C for 48 h. The plates were washed with PBS-T after incubation with biotinylated an anti-mouse IFN-γ or IL-4 antibody overnight at 4 °C. After washing, streptavidin-AP was added to each well and incubated for 2 h at room temperature. After washing with PBS-T, the plates were incubated with a BCIP/NBT solution for 30 minutes at room temperature. Finally, the plates were rinsed with water and air-dried at room temperature, and colored spots were counted using a dissecting microscope (Leica LMD6500, Leitz-Park, Germany).

### 2.9. Statistical Analysis

All data are expressed as the mean ± SEM. Differences between 2 groups were assessed using an unpaired 2-tailed Student’s *t* test. Datasets involving more than 2 groups were assessed with Tukey’s post hoc test using Prism version 5.01 (GraphPad Software. San Diego, CA). Survival curves were analyzed using the Kaplan–Meier method with a log-rank test. *p* < 0.05 was considered significant.

## 3. Results

### 3.1. Screening of Appropriate Antigen Sequence for IL-17A DNA Vaccine

**Figure 1:**
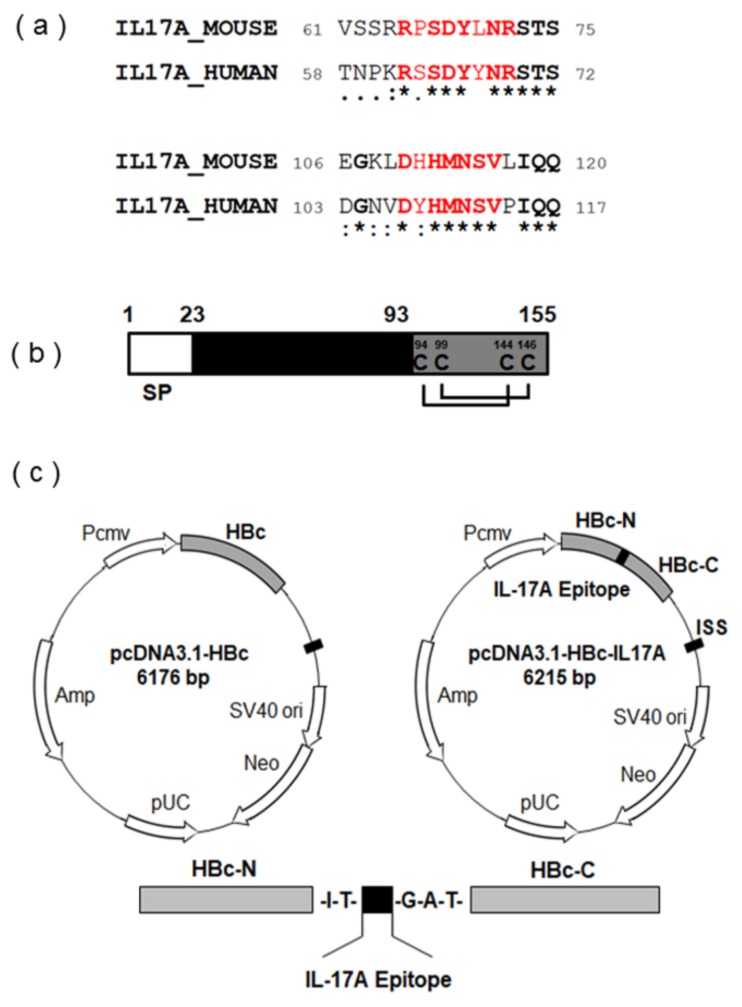
Plasmid DNA construction for IL-17A vaccination; (**a**) BLAST alignment for mouse and human IL-17A of two selected B cell epitope sequences, 17A1 (upper) and 17A2 (lower), compared based on sequence alignment using GENETYX (Software Development, Tokyo, Japan) and homology analysis using BLAST programs from the GenBank database. Epitope sequences of the IL-17A DNA construct are shown in red. Bold letters with asterisk (*) indicate regions where the two species have an identical amino acid; period (.) indicates weak similarity and colon (:) indicates strong similarity, while regions with conserved changes are indicated by no signature. Amino acid (aa) sequences are included with the numbers that represent the aa positions of each peptide. (**b**) Scheme of mouse IL-17A protein. Signal peptide (SP; aa 1–23), mature protein (aa 23–93), and two disulfide bonds (cysteine; aa 94–144 and 99–146) are indicated. (**c**) Construction of IL-17A DNA vaccines. Left panel: Plasmid map of pcDNA3.1-HBc. HBc gene was cloned downstream of CMV promoter. Right panel: Plasmid map of pcDNA3.1-HBc-IL-17A. DNA fragment encoding IL-17A epitope (17A1, 17A2) and its N- and C-terminal linkers were inserted at positions corresponding to amino acids 80–81 of hepatitis B core (HBc). Lower panel: Schematic representations of fusion protein HBc-IL-17A epitope construction. Oligonucleotide sequences of a dipeptide spacer, Ile-Thr, or a tripeptide spacer, Gly-Ala-Thr, were introduced at the N- or C-terminus of the IL-17A epitope, respectively. Coding region for the IL-17A epitope (17A1, 17A2) was inserted into the immunodominant region (aa 80–81) of HBc. Cloned IL-17A epitope sequences were designed to allow for conformational flexibility of spacers when the surface is exposed on the HBc particle. Spacers are represented by single letter codes.

The IL-17 cytokines make dimeric proteins, either homodimeric or heterodimeric forms. IL-17A, IL-17F, and IL-17A/F require a heterodimeric receptor complex comprising IL-17RA and IL-17RC for signaling. We narrowed down our candidates to IL-17A, because IL-17RA has 100-fold higher affinity to IL-17A than to IL-17F among the six IL-17 family members [11]. As an initial step, we predicted the candidate sequence for B cell epitope against mouse IL-17A based on the BepiPred-2.0 system (Department of Health Technology, Lyngby, Denmark; http://www.cbs.dtu.dk/services/BepiPred/). Furthermore, we confirmed the location of these selected epitopes based on the predicted three-dimensional structure (Swiss-model: https://swissmodel.expasy.org/). Two target B cell epitopes for mouse IL-17A, 17A1 (amino acids 65–72), and 17A2 (amino acids 110–116) were selected. BLAST alignment including the candidate B cell epitope for 17A1 or 17A2 showed that these epitopes did not include identical residues between IL-17A and IL-17F but did include residues unique to IL-17A. In addition, these amino acid sequences were highly conserved between mouse IL-17A (accession #Q62386) and human IL-17A (accession #Q16552), according to the analysis using BLAST programs from the GenBank database (Figure 1a). Furthermore, these selected epitopes were not located in the receptor binding interface on the three-dimensional structure of the receptor complex, which adopts many forms with an IL-17A dimer. IL-17A forms a dimeric complex with IL-17A or IL-17F using two disulfide bond regions, C^94^–C^144^ and C^99^–C^146^ (Figure 1b), and the 17A2 epitope sequence shares the disulfide bond regions (cysteine, amino acids 94–144 and 99–146).

**Figure 2:**
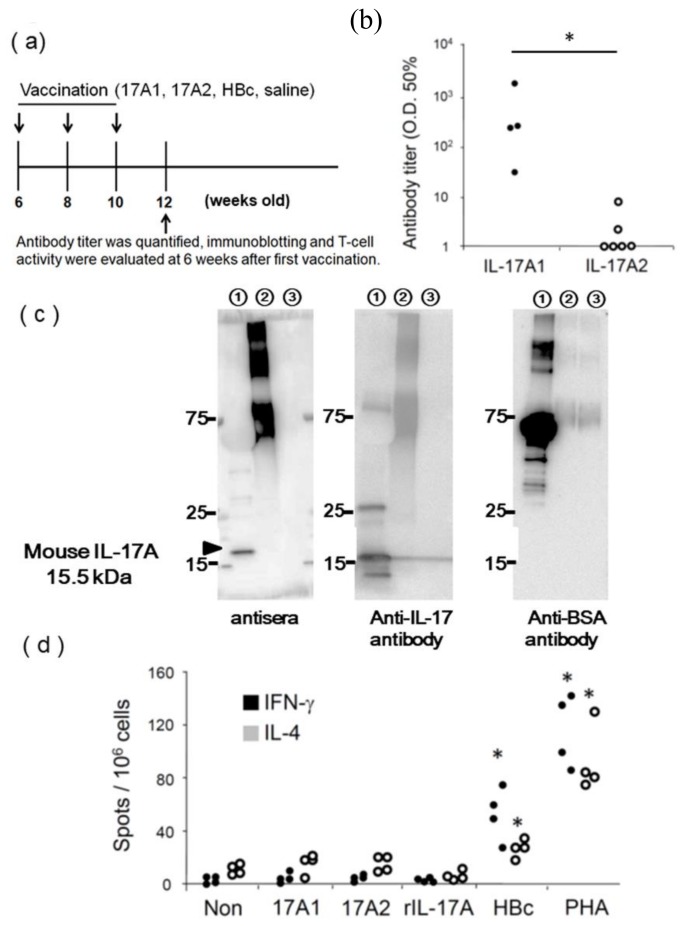
Evaluation of anti-IL-17A antibodies induced by IL-17A vaccination in BALB/c mice; (**a**) Female BALB/c mice were immunized with pcDNA3.1-HBc-IL17A1 (IL-17A1), pcDNA3.1-HBc-IL17A2 (IL-17A2), pcDNA3.1-HBc (HBc), or saline via intramuscular administration using an electroporator three times at two week intervals. We collected serum from immunized mice, and antibody titers were measured by ELISA at 6 weeks after the first vaccination (12 weeks old). Immunoblotting and T-cell activity assays were also performed using serum from 12-weeks-old mice. (**b**) The anti-IL-17A1 antibody titer (OD 50 %) was increased, but the anti-IL-17A2 titer was not (*n* = 6). (**c**) (left panel) Antisera from IL-17A1 immunized mice detected an IL-17A1-BSA conjugate and recombinant mouse IL-17A, but not an IL-17A2-BSA conjugate. (middle panel) A commercially available anti-IL-17A antibody also detected recombinant mouse IL-17A. (right panel) An anti-BSA antibody detected BSA, but not recombinant mouse IL-17A. Lane 1, recombinant mouse IL-17A with BSA; lane 2, IL-17A1-BSA conjugated peptide; and lane 3, IL-17A2-BSA conjugated peptide. (**d**) In an ELISPOT assay, splenocytes (10^6^ cells per well) from IL-17A1-immunized mice at 12 weeks of age were stimulated with 17A1 peptide, 17A2 peptide, recombinant mouse IL-17A (rIL-17A), HBc peptide, or PHA at 10 μg/mL. The production of IFN-γ or IL-4 by splenocytes was detected as black spots. The splenocytes of six mice were tested in the ELISPOT assay. The number of spots was quantified in triplicate wells of each group. * *p* < 0.05 vs no stimulation.

As a carrier protein, HBc is known to self-assemble into icosahedral virus-like particles (VLPs) [12]. The DNA fragment of the mouse IL-17A epitopes (17A1 and 17A2) was inserted into the position corresponding to the B cell epitope (amino acid 80–81) of HBc (Figure 1c). The resulting plasmid, pcDNA3.1-HBc-IL-17A, can be utilized as a DNA vaccine with an adjuvant effect, because plasmid DNA itself is known to have adjuvant effects. To confirm whether this DNA vaccine system would sufficiently induce anti-IL-17A1 or anti-IL-17A2 antibody production, female BALB/c mice were immunized with pcDNA3.1-HBc-IL17A1 (17A1), pcDNA3.1-HBc-IL17A2 (17A2), pcDNA3.1-HBc (HBc), or saline via intramuscular administration using an electroporator three times every 2 weeks (Figure 2a). We confirmed that the titer of anti-IL-17A1, but not that of anti-IL-17A2, was increased 6 weeks after the first vaccination (Figure 2b). Furthermore, serum from IL-17A1-immunized mice (antiserum) was utilized as a primary antibody for Western blotting (Figure 2c). The antisera detected a 15.5 kDa band with recombinant IL-17A protein (lane 1 in the Figure 2c) and a greater than 75 kDa band with BSA-conjugated IL-17A1 peptide (lane 2 in the Figure 2c), but not with BSA-conjugated IL-17A2 peptide (lane 3 in the Figure 2c). The pattern produced by the antisera was similar to that produced by a polyclonal anti-IL-17A antibody (Figure 2c). An anti-BSA antibody detected an approximately 66 kDa band with BSA (lane 1), IL-17A1-BSA (lane 2 in the Figure 2c), and IL-17A2-BSA (lane 3 in the Figure 2c) because BSA was added as a carrier protein in commercially available recombinant IL-17A (Figure 2c). However, the serum from IL-17A2-immunized mice 6 weeks after vaccination did not detect recombinant IL-17A protein or BSA-conjugated IL-17A2 (data not shown). Although the mice immunized with the 17A2 immunogen did not exhibit high levels of antibody against 17A2, the antibody titer against 17A1 in mice immunized with the 17A1 immunogen stabilized approximately 6 weeks after the first vaccination. Therefore, the 17A1 epitope was used in subsequent experiments throughout the immunization protocol.

To evaluate the activation of T cells, we examined the antigen reactivity of splenocytes from IL-17A1-immunized mice using an ELISpot assay. T cell activity was evaluated 6 weeks after the first vaccination. Splenocytes from IL-17A1-immunized mice were significantly stimulated by the HBc epitope peptide and PHA but not by the IL-17A1 epitope, the IL-17A2 epitope, or recombinant IL-17A (Figure 2d and Figure S1), which suggest that only HBc contains a T cell epitope. Therefore, the IL-17A1 epitope could activate B cells but not T cells in our animal model. This situation is reflected in the relationship between hapten and its carrier, in which hapten has only a B cell epitope and the carrier possesses a T cell epitope. Based on this finding, we used IL-17A1 epitope and HBc as hapten and its carrier to successfully produce an antibody against IL-17A, which was assisted by helper T cell activation. The results indicate that this IL-17A vaccine did not induce autoreactive T cells against endogenous IL-17A. Therefore, we selected the sequence of the IL-17A1 epitope as the IL-17A DNA vaccine and examined the therapeutic effects of the vaccine in SLE model mice.

### 3.2. Evaluation of IL-17A1 Vaccine in NZBWF1 Mice

**Figure 3:**
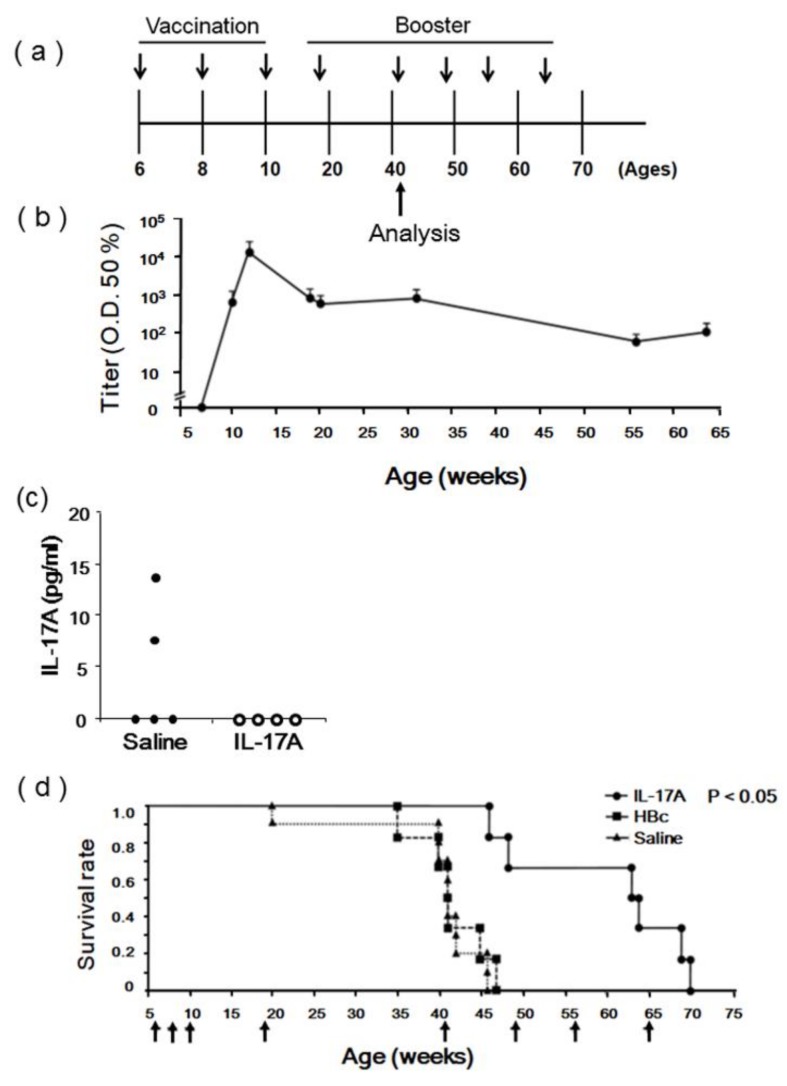
Effects of IL-17A DNA vaccine in NZBWF1 mice; (**a**) NZBWF1 mice were immunized with pcDNA3.1-HBc-IL17A1 (IL-17A1), pcDNA3.1-HBc (HBc), or saline via intramuscular administration using an electroporator three times at 2-week intervals. Booster immunization was also performed at 19-, 41-, 49-, 56-, and 64- weeks old. NZBWF1 mice immunized with IL-17A1 vaccine were analyzed 34 weeks after the first vaccination (approximately 40 weeks old); (**b**) Sustained high-titer antibody responses induced by IL-17A vaccine. Anti-IL-17A antibody titer assessed by ELISA is expressed as serum dilution that exhibited half-maximal binding in the NZBWF1 mice. It was measured 4-, 6-, 13-, 14-, 25-, 50-, and 58- weeks after the first vaccination (*n* = 8); (**c**) Serum IL-17A concentration was decreased in the IL-17A vaccine group 25 weeks after the first vaccination; *n* = 5 and 4, respectively; (**d**) Kaplan–Meier curve of time to survival rate, log rank *p* < 0.05 between HBc-IL-17A group and nonimmunization groups (HBc and saline). The arrows indicate vaccination. (●)—IL-17A vaccine group (*n* = 6); (■)—HBc vaccine group (*n* = 6); (▲)—saline group (*n* = 10).

To evaluate the therapeutic effects of the IL-17A DNA vaccine, we selected female NZBWF1 mice, which spontaneously develop an autoimmune disease closely resembling human SLE. By 40 weeks of age, more than 90% of NZBWF1 mice develop glomerulonephritis [13,14]. Female NZBWF1 mice were immunized with pcDNA3.1-HBc-IL17A1 (IL-17A), pcDNA3.1-HBc (HBc), or saline via intramuscular administration using an electroporator three times every 2 weeks and were analyzed at approximately 40 weeks old. A booster immunization was performed in 19-, 41-, 49-, 56-, and 64-week-old mice to maintain the anti-IL-17A antibody titer (Figure 3a). We confirmed that immunized IL-17 DNA vaccines also induced anti-IL17A antibody production in NZBWF1 mice. The number of antibodies stabilized around a maximal titer 6 weeks after the first vaccination and remained stable until the end of the experiment (Figure 3b). Although the serum IL-17A concentrations were increased in NZBWF1 mice [13], the serum IL-17A concentration was significantly reduced in the IL-17A-vaccinated mice 25 weeks after the first vaccination (Figure 3c) even though it showed large individual differences. Importantly, the survival rate of NZBWF1 mice was significantly increased in the IL-17A vaccine group (Figure 3d). Thus, the DNA vaccine for IL-17A significantly improved survival while sustaining anti-IL-17A antibody titers in lupus-prone mice.

**Figure 4:**
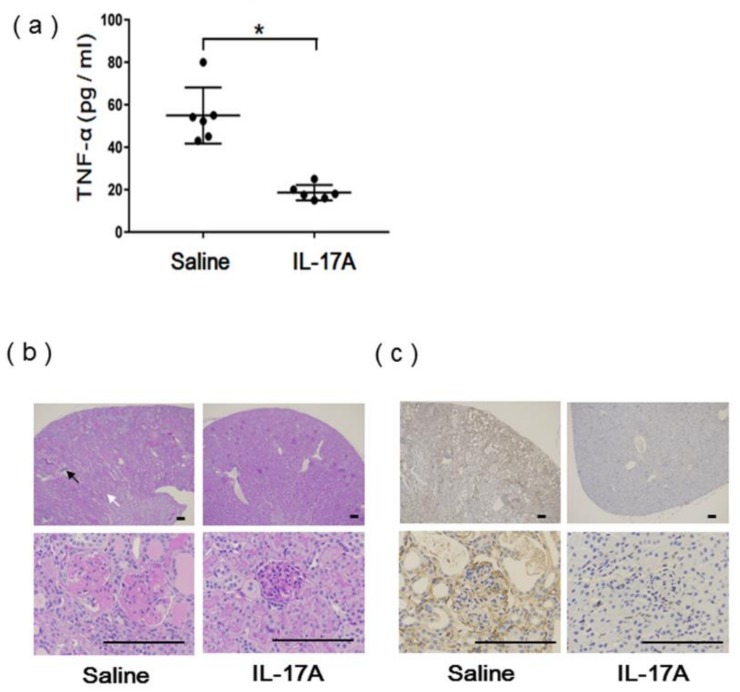
Serum cytokines in vaccinated NZBWF1 mice; (**a**) Serum TNF-α concentration was significantly decreased in IL-17A vaccine group 34 weeks after the first vaccination; * *p* < 0.05 vs. saline group; *n* = 5. (**b**) Periodic acid Schiff (PAS) staining of kidney sections from IL-17A vaccine group (right) and saline group (left). Glomerulosclerosis, glomerular necrosis, tubular atrophy (white arrow), and vasculitis (black arrow) were suppressed in IL-17A vaccine group 36 weeks after first vaccination. Scale bar = 100 μm. (**c**) F4/80 immunostaining of kidney sections from IL-17A vaccine group (right) and saline group (left). Infiltration of macrophages was suppressed in IL-17A vaccine group 36 weeks after first vaccination. Scale bar = 100 μm.

The levels of serum TNF-α from the same mice were significantly decreased in the IL-17A-vaccinated mice (Figure 4a). This finding is consistent with a previous report that the systemic serum concentration of tumor necrosis factor (TNF-α) in NZBWF1 mice was significantly increased at greater than 30 weeks of age [13]. According to the pathological analysis, glomerular necrosis, glomerulosclerosis, interstitial infiltration, tubular atrophy, vasculitis, and macrophage infiltration, shown by staining with an anti-F4/80 antibody, were dramatically reduced in the IL-17A vaccine group compared to the saline group 36 weeks after vaccination (Figure 4b,c). Additionally, submandibular sialadenitis, which has been known to develop in NZBWF1 mice [13], was suppressed in the IL-17A vaccine group 36 weeks after vaccination (Figure S2a). To assess safety, H&E staining of the liver and spleen 36 weeks after vaccination showed no damage or immune cell infiltration in the IL-17A vaccine group (Figure S2b,c). These results suggest that IL-17A DNA vaccine-induced antibodies successfully attenuated the function of IL-17A in renal dysfunction and submandibular sialadenitis through decreased inflammatory cytokine production and macrophage infiltration.

### 3.3. Evaluation of IL-17A1 DNA Vaccine in MRL/lpr Mice

**Figure 5:**
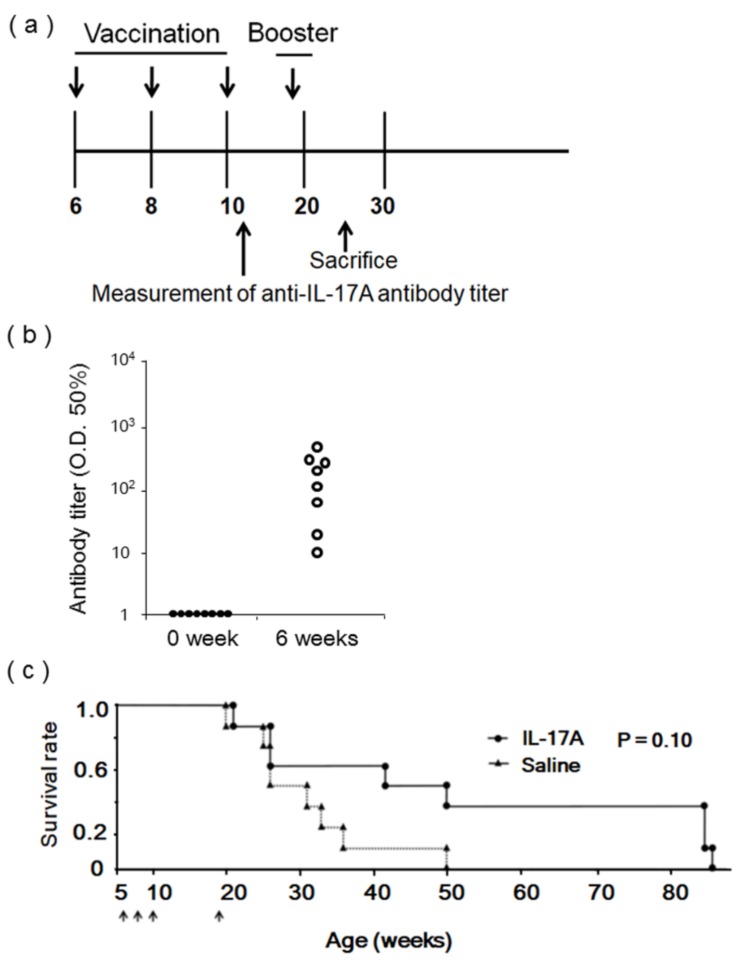
Effects of IL-17A DNA vaccine in MRL/lpr mice; (**a**) Female MRL/lpr mice were immunized with pcDNA3.1-HBc-IL17A1 (IL-17A1) or saline via intramuscular administration using an electroporator three times at 2-week intervals. Booster immunization was performed 13 weeks after three vaccinations. Antibody titers of vaccinated MRL/lpr mice were measured 6 weeks after first vaccination (12 weeks old), and mice were sacrificed 18 weeks after first vaccination (24 weeks old). (**b**) Antibody titers from vaccinated 12-week-old MRL/lpr mice were measured (*n* = 8). (**c**) Time course of IL-17A DNA vaccination for survival experiments. Kaplan–Meier curve of time to survival rate, log rank *p* = 0.10 between HBc-IL-17A group and nonimmunization group (saline). The arrows indicate vaccination. (●): IL-17A vaccine group (*n* = 6); (▲): saline group (*n* = 8); The arrows indicate the intramuscular administration of Il-17A vaccine or Saline using an electroporator.

Female MRL/lpr mice, another SLE model [15], were also immunized with pcDNA3.1-HBc-IL17A1 (IL-17A) or saline as a control via intramuscular administration using an electroporator three times every 2 weeks. Antibody titers were quantified 6 weeks after vaccination (12 weeks old), and booster immunization was performed 12 weeks after the first vaccination (18 weeks old) (Figure 5a). Similarly, the anti-IL-17A antibody titer was significantly increased 6 weeks after the first vaccination in MRL/lpr mice (Figure 5b). Serum TNF-α was significantly reduced in IL-17A vaccine immunized same MRL/lpr mice (Figure 5c).

**Figure 6:**
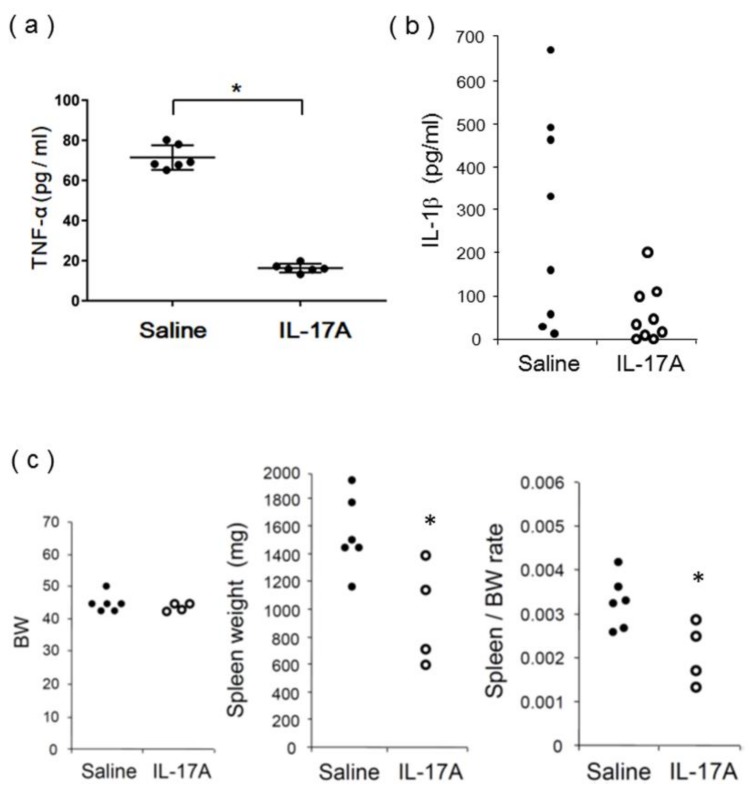
Serum cytokines and spleen weight in vaccinated MRL/lpr mice; (**a**) Serum TNF-α concentration was significantly decreased in IL-17A vaccine group; * *p* < 0.05 vs. saline group; *n* = 5. (**b**) Serum IL-1β concentration was significantly decreased in IL-17A vaccine group; * *p* < 0.05 vs. saline group; *n* = 8 and 9, respectively. (**c**) Spleen/body weight ratio was significantly decreased in vaccine group; * *p* < 0.05 vs. saline group; *n* = 6 and 4, respectively.

In the pathological findings, glomerulosclerosis, interstitial infiltration, and macrophage infiltration were significantly attenuated in IL-17A vaccine-immunized MRL/lpr mice 18 weeks after vaccination (Figure S3a,b). Consistently, serum TNF-α and IL-1β levels were significantly reduced in IL-17A vaccine-immunized MRL/lpr mice (Figure 6a,b). H&E staining of the liver and spleen 18 weeks after vaccination showed no damage or immune cell infiltration in IL-17A vaccine-immunized MRL/lpr mice (Figure S3c,d). To investigate the inhibitory effects of the vaccine against splenomegaly, a manifestation of SLE, the spleen/body weight (BW) ratio was determined in 24-week-old MRL/lpr mice. Since splenomegaly is associated with disease progression in MRL/lpr mice [15], the body and spleen weights of the immunized mice were determined 18 weeks after vaccination (Figure 6c). There was no difference in BW between vaccinated and nonvaccinated MRL/lpr mice. In contrast, the spleen weight and spleen/BW ratio were significantly decreased in IL-17A vaccine-immunized MRL/lpr mice. These results suggest that the IL-17A DNA vaccine-induced antibodies successfully attenuated the role of IL-17A in MRL/lpr mice.

As there are two amino acid differences between the mouse IL-17A1 and human IL-17A1 epitopes (Figure 1a), we evaluated the immunogenicity of the human IL-17A1 epitope in mice. Female BALB/c mice were immunized with pcDNA3.1-HBc-human IL17A1 (human IL-17A) via intramuscular administration using an electroporator three times every 2 weeks. As a result, anti-human IL-17A1 epitope antibodies were produced in mice 6 weeks after the first vaccination, and the produced antibodies reacted more weakly to the mouse IL-17A1 epitope sequence than to the human IL-17A1 epitope sequence (Figure S4). These results indicate that vaccination with the IL-17A1 epitope sequence might be useful for therapeutic approaches in IL-17-mediated autoimmune disease. At least, it will be expected that the antibody titer against human IL-17A1 will be successfully increased in mice.

## 4. Discussion

In this study, an IL-17A therapeutic DNA vaccine successfully attenuated the progression of lupus nephritis in SLE model mice, indicating that IL-17A could be a good therapeutic target for SLE. In recent reports concerning its function in autoimmune diseases, IL-17A has been shown to drive autoimmune responses by promoting the formation of spontaneous germinal centers [16] and the production of autoantibodies.

In terms of the functional importance of IL-17A in SLE and lupus nephritis, the induction of SLE by pristane in IL-17-sufficient wild-type mice did not occur in IL-17A-deficient mice, which were protected from the development of lupus autoantibodies and glomerulonephritis [17]. The role of IL-17A in SLE has also been addressed in Fcgr2b^-/-^ mice [18]. The lack of this inhibitory receptor is responsible for the loss of B cell tolerance, leading to autoantibody formation and fatal lupus-like glomerulonephritis. By using Fcgr2b^-/-^ mice that were also deficient in Act1, a crucial adaptor molecule downstream from the IL-17RA/IL-17RC complex, it was shown that IL-17A deletion significantly reduced inflammatory monocyte and neutrophil infiltration and thus the severity of glomerulonephritis. These reports using IL-17A-deficient mice suggest that neutralization of IL-17A signaling by small molecules or vaccines may serve as a novel therapy to treat lupus nephritis and SLE.

To attenuate the function of IL-17A, we proposed a therapeutic DNA vaccine to induce anti-IL-17A antibodies. Although a peptide vaccine using full-length IL-17A as an immunogen was reported [19], cytotoxic T cells might attack the cells expressing IL-17A and cause harmful side effects, since the full-length IL-17A used for immunization induced cellular immunity against IL-17A. In addition, antibodies showing cross-reactivity with other cytokines similar to IL-17A may also be produced. Therefore, a vaccine using only a partial epitope of IL-17A with no homology with other proteins as an immunogen should be considered to accommodate the safety perspective. Although two types of IL-17A epitope vaccines have been previously tested in inflammatory bowel disease model mice [20], those vaccines failed to show an improvement in the pathology. These results strongly suggest that the selection of an appropriate epitope is quite important to develop therapeutic vaccines.

In contrast, the IL-17A epitope DNA vaccine successfully produced anti-IL-17A antibodies and ameliorated the symptoms of SLE in our study. The selected IL-17A epitope was short enough to avoid activation of a cytotoxic autoimmune reaction. These findings indicate that the IL-17A epitope DNA vaccine shown in this study is safe and effective. Additionally, the serum IL-17 concentrations in vaccinated and nonvaccinated lupus-prone mice were measured by ELISA. The alterations of serum IL-17 after vaccination were not significantly different because the plasma IL-17A concentration was not as high as in spontaneous lupus-prone mice [21]. In this study, we utilized two mouse strains, NZBWF1 and MRL/lgr, as models of SLE. The MRL/lgr mouse strain has shown more rapid and severe disease development than the NZBWF1 mouse strain [22,23]. We speculate that the different responses to the IL-17A vaccine between the two strains might be due to the severity of the disease model. However, in the analysis of the MRL/lgr mouse strain, we confirmed the same tendency to attenuate disease progression, although the effect was less than that in the NZBWF1 mouse strain. IL-17A has been known to play an important role in the aggravation of pathology, not only in several cancers but also in autoimmune diseases such as rheumatoid arthritis, spondyloarthritis, multiple sclerosis, and psoriasis [24]. A limitation of this study was that it evaluated the effect of an IL-17A DNA vaccine in a preventive model, but not in a progressive model of disease. This advance will be examined in the near future toward clinical application. In addition, there were different injection protocols for MRL/lgr and NZBWF1 mice in this study. The suitable usage schedule has been fixed for the next trial.

Recently, it has been reported that anti-IL-17A antibody drugs show remarkable therapeutic effects in psoriasis patients [25]. However, antibody drugs are very expensive, and autoantibodies to these drugs are produced with continuous use, which might impair their efficacy (secondarily ineffective). Therefore, a vaccine targeting IL-17A would be effective for these diseases and may provide an effective therapeutic agent at low cost over a long term.

## 5. Conclusions

Taken together, these data show that immunization with our selected IL-17A epitope DNA vaccine successfully produced anti-IL-17A antibodies, significantly reduced organ damage, and extended survival time in lupus-prone mice. In the future, development of an IL-17A vaccine may provide a novel therapeutic strategy to treat SLE and other autoimmune disorders, such as rheumatoid arthritis, spondyloarthritis, and psoriasis.

## Supplementary Materials

The following are available online at https://www.mdpi.com/2076-393X/8/1/83/s1, Figure S1: ELISPOT assay of IL-17A immunized mice; Figure S2: H&E staining of submandibular grand, liver and spleen from IL-17A vaccinated or saline-treated NZBWF1 mice; Figure S3: Pathological analysis of Il-17A vaccinated or saline-treated MRL/lpr mice; Figure S4: Evaluation of human IL-17A epitope.
