# Supplementary material for: Development of an IL-17A DNA Vaccine to Treat Systemic Lupus Erythematosus in Mice
Source: Vaccines (Basel). 2020 Feb 12;8(1):83. doi: 10.3390/vaccines8010083 (PMC7157613; doi:10.3390/vaccines8010083)
Supplement: Supplementary file 1 [file vaccines-08-00083-s001.pdf]

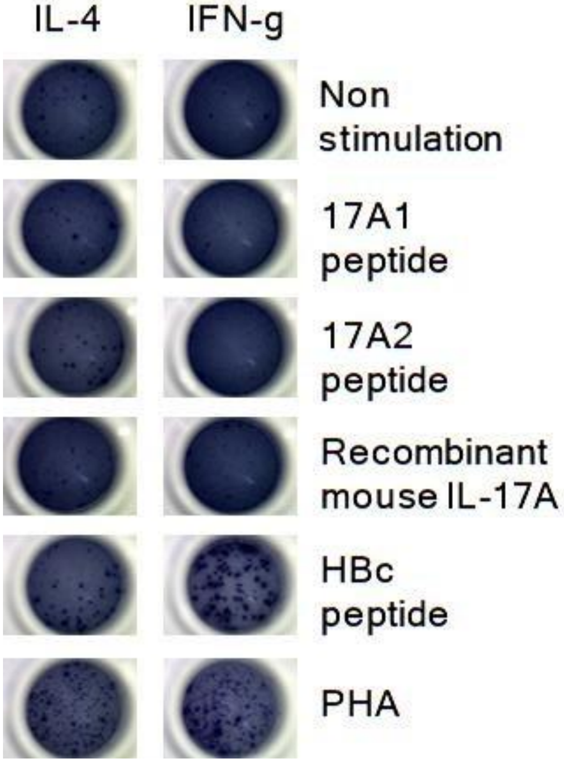

Figure S1. ELISPOT assay of IL-17A immunized mice. In the ELISPOT assay, splenocytes (10^6 cells per well) from IL-17A1- immunized mice at 12 weeks old were stimulated with 17A1 peptide, 17A2 peptide, recombinant mouse IL-17A (rIL-17A), HBc peptide, or PHA at 10 μg/mL. The production of IFN-γ or IL-4 by splenocytes was detected as black spots. The splenocytes of six mice were tested in ELISPOT assay, respectively.

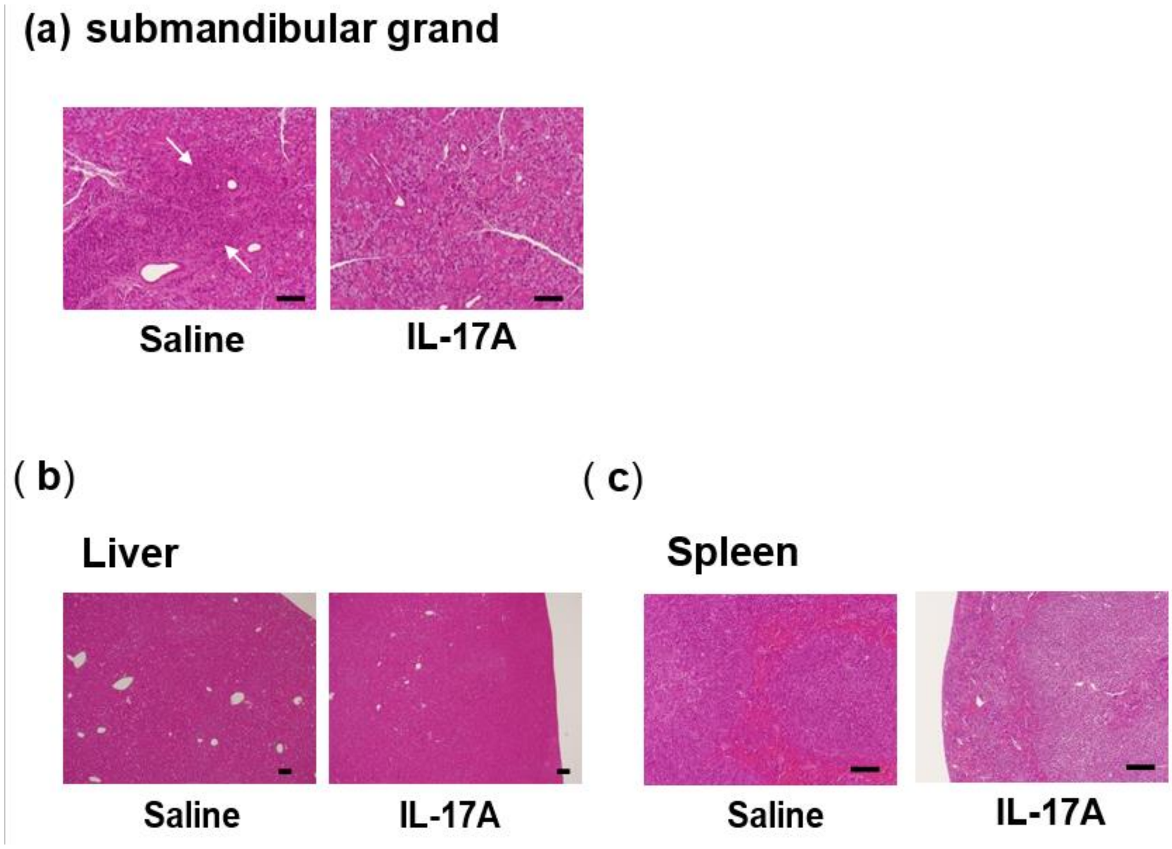

Figure S2. H&E staining of submandibular grand, liver and spleen from IL-17A vaccinated or saline-treated NZBWF1 mice. (**a**) H&E staining of submandibular grand section of the IL-17A vaccine group (right) and saline group (left). Submandibular sialadenitis was suppressed in the IL-17A vaccine group. White arrows indicate destruction of the normal structures of submandibular grand by dense lymphoid cell infiltration. Scale bar = 100 mm. (**b, c**). There was no evidence of any pathological changes in vaccinated NZBWF1 mice. (**b**) liver section, (**c**) spleen section in IL-17A vaccine group (right) and saline group (left). Scale bar = 100mm.

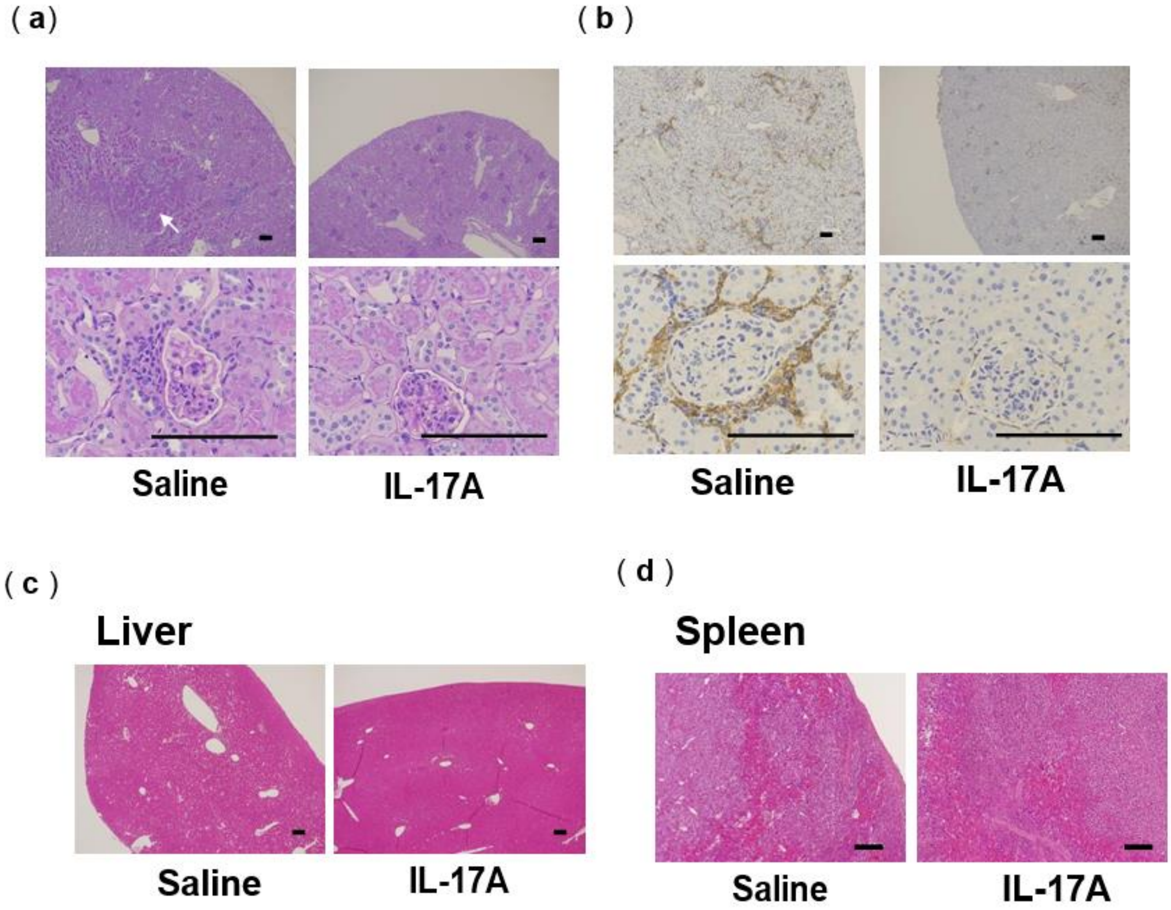

Figure S3. Pathological analysis of Il-17A vaccinated or saline-treated MRL/lpr mice. (**a**) PAS staining of kidney sections from the IL-17A vaccine group (right) and the saline group (left). Glomerulosclerosis and interstitial infiltration (white arrow) were suppressed in the IL-17A vaccine group. Scale bar=100 μm. (**b**) F4/80 immunostaining of kidney sections from the IL-17A vaccine group (left) and the saline group (right). Infiltration of macrophages was suppressed in the IL-17A vaccine group. Scale bar = 100 μm; (**c**,**d**) HE staining of liver and spleen section of IL-17A vaccine group (right) and saline group (left). There was no evidence of any pathological changes in tissue sections of vaccinated MRL/lpr mice. Scale bar = 100μm.

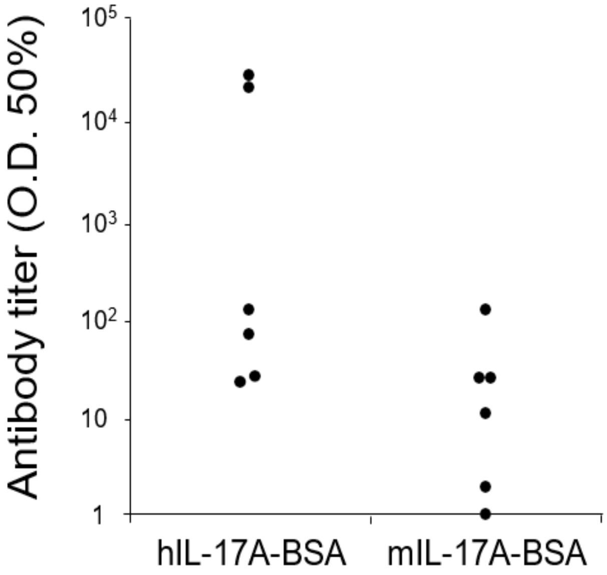

Figure S4. Evaluation of human IL-17A epitope. Balb/c female mice were immunized with pcDNA3.1-HBc- humanIL17A1 (human IL-17A) three times every two weeks. Anti- humanIL-17A1 epitope antibody was produced at 6 week after first vaccination (hIL-17A-BSA, left), and produced antibody cross-reacted weakly to mouseIL-17A1 epitope (mIL-17A-BSA, right).
